# The Potential for Large Language Models in Eating Disorder Care: Opportunities for Education, Detection and Assessment, and Intervention

**DOI:** 10.1007/s11920-026-01721-0

**Published:** 2026-10-27

**Authors:** Jake Linardon

**Affiliations:** School of Psychology, Faculty of Health, Deakin University, 75 Pigdons Road, Geelong, Victoria 3216 Australia

**Keywords:** Eating disorders, Large language models, Artificial intelligence, Psychiatry

## Abstract

**Purpose of Review:**

Large language models (LLMs) are anticipated to transform the eating disorder field, offering new opportunities for education, detection and assessment, and intervention. This paper synthesizes evidence on these emerging opportunities and sets a future research agenda.

**Recent Findings:**

Early research provides encouraging evidence that LLMs have clinically relevant capabilities in eating disorders. For education, LLMs can provide accurate responses to consumer-facing eating disorder queries, although performance varies by topic complexity. For detection and assessment, LLMs can identify probable diagnoses from vignettes and translate online discourse into risk signals. For intervention, LLMs are capable of providing empathic support and caregiver guidance, but can also generate harmful outputs.

**Summary:**

LLMs offer opportunities to expand access to eating disorder support. The evidence base, safeguards, and evaluation standards established now will determine whether these tools can be used safely in practice, necessitating the need for further research in clinical settings.

## Introduction

Eating disorders are prevalent illnesses that impair functioning and are associated with high rates of mortality and psychiatric comorbidity [1]. Despite considerable progress in establishing valid diagnostic instruments and evidence-based interventions, most people with eating disorders remain undetected and untreated [2]. This gap is driven not only by patient-level barriers such as prohibitive costs, stigma, and geographic isolation, but also by a critical workforce deficit in which demand for eating disorder care far exceeds the availability of clinicians trained to provide it [3]. Even within specialist services, clinicians face considerable administrative burden that limits the time available for direct patient-facing care.

Large language models (LLMs) are breakthrough technologies that could help to address this widespread service gap. Trained on vast datasets, LLMs can interpret free-text input and generate fluent, contextually responsive language [4]. These models can be broadly distinguished as general-purpose systems (e.g., ChatGPT, Claude, and Gemini), which are designed to perform a wide range of tasks, and domain-specific systems, which adapt general-purpose LLMs for a narrower clinical use case through fine-tuning or curated knowledge bases. Given their scalability and capacity to simulate personalized dialogue, LLMs may be well suited to supporting selected aspects of mental health care, including helping users better understand their emotional distress, assisting therapists with case formulation or treatment planning, identifying relevant support options, recommending appropriate self-management strategies, or providing a supportive space for users to reflect on their mental health difficulties [5–8]. Critically, these capabilities may extend across the continuum of mental healthcare, from identifying emerging risk and delivering self-guided preventive support, to augmenting treatment for established illness.

LLMs are anticipated to become integrated into the eating disorder care landscape. Preliminary data already suggest early uptake, with nearly two-thirds of sampled eating disorder clinicians reporting use of general-purpose LLMs for administrative purposes and one-sixth of community participants reporting use for education or self-help [9]. These data also suggest cautious optimism about the role of LLMs in eating disorder care, with participants expressing openness to their use and acknowledging their potentially beneficial role in patients’ care plans, while also raising concerns about privacy, over-reliance, and adequate training [9]. However, before LLMs become routinely embedded in eating disorder care, rigorous research is needed to determine the scope of their capabilities and the contexts in which they add genuine clinical value. Without this evidence, technologies intended to improve care access risk providing misleading guidance or deepening existing inequities in patient care.

**Fig. 1:**
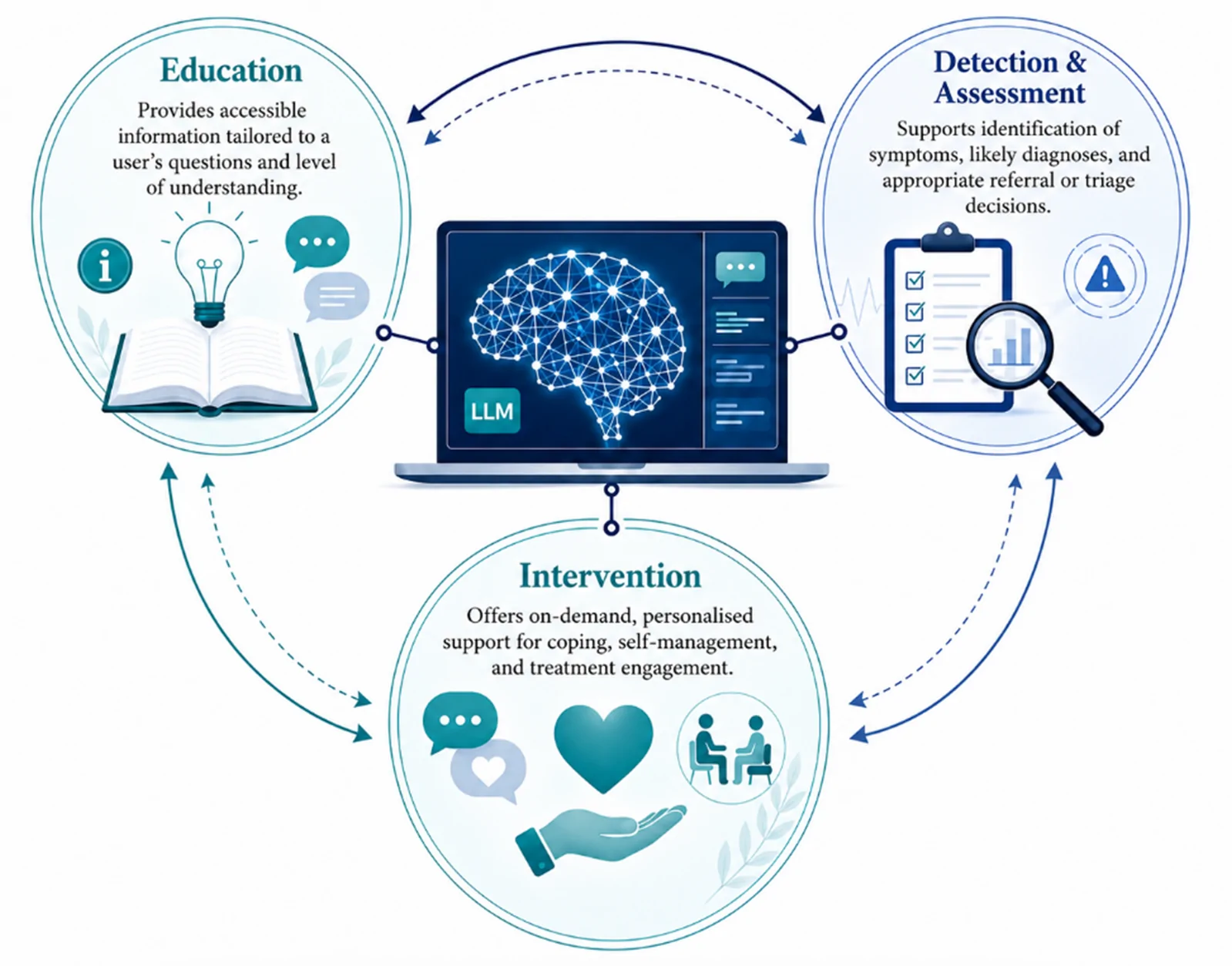
Core clinical capabilities of large language models in mental health and eating disorder care

This paper discusses the core capabilities of LLMs in mental health care and reviews the nascent literature on their clinical potential and risks in eating disorders, organized around the broad domains of education, detection and assessment, and intervention (Fig. 1). The capabilities discussed are relevant across the full spectrum of care, from prevention and early intervention through to treatment, because the same functions may support individuals with emerging risk as well as those with established, clinically significant symptoms. By mapping the current evidence base and highlighting key literature gaps, it aims to provide a roadmap for the next phase of research needed to determine whether LLMs can deliver meaningful added value to eating disorder care.

## Education

One of the clearest opportunities for LLMs is their capacity to deliver education about mental health. By instantaneously responding to user queries in natural language and adapting explanations to a person’s level of comprehension, LLMs can reduce the burden of searching through and interpreting static mental health information. This form of low-cost, widely accessible education could strengthen mental health literacy at scale and potentially reduce stigma, improve symptom recognition, and ultimately encourage help-seeking [10]. It may also hold value for workforce development by supporting trainees and non-specialist clinicians with on-demand education.

However, these downstream benefits are predicated on LLMs being able to provide information that remains factually accurate and clinically appropriate when applied to specialized mental health domains. If these standards are not met, educational outputs that appear authoritative risk reinforcing misconceptions about mental illness or delaying access to appropriate clinical care. Emerging research suggests that general-purpose LLMs can answer psychiatry knowledge items with fairly high accuracy [11], especially for newer model iterations and lower-complexity topics [12], and that clinician-assisted use of ChatGPT may improve answer accuracy, completeness, and nuance in some psychiatry tests [13]. Domain-specific language models may perform even better on educationally relevant tasks. For example, CBT-LLM, which was purposively trained on a Chinese mental health question-answer dataset structured around cognitive-behavioral therapy principles, outperformed non-trained comparator models on human-rated relevance, CBT-consistent structure, and helpfulness [14]. Similarly, the language model PsycoLLM, trained on psychological question-answer, dialogue, and knowledge-based datasets, achieved stronger performance than comparator models on benchmark assessments of professional ethics and theoretical knowledge [15].

Less is known about whether LLMs can generate clinically appropriate educational outputs in the context of eating disorders. The limited research available provides encouraging early signals but remains largely proof-of-concept evaluations in which general-purpose LLMs are tested on relatively small sets of researcher-generated prompts. The clearest example of LLMs’ capacity to generate appropriate educational outputs about eating disorders was demonstrated by Yeşilkaya et al. [16], who prompted ChatGPT-4o, Gemini, and DeepSeek with 20 standardized questions typically asked of parents of adolescents with anorexia nervosa, spanning early recognition, diagnosis, clinical management, and long-term outcomes. The authors observed high levels of accuracy across models overall (88–92%) alongside strong reproducibility across repeated prompts (83–90%). Performance was generally highest for items relating to treatment approaches and long-term prognosis, and ChatGPT produced the fewest factual errors overall (*N* = 3), compared with Gemini (*N* = 5) and DeepSeek (*N* = 7). These findings suggest that widely used LLMs can provide accurate information for consumers seeking guidance about anorexia nervosa, although errors do persist and performance appears sensitive to question complexity. Whether these results generalise to other eating disorder presentations or educational contexts remains poorly understood.

Beyond answering questions, newer LLMs are capable of generating images from virtually any natural-language prompt, opening another way these tools may influence public understanding of eating disorders. Recent research illustrates this by documenting how LLMs portray eating disorders through image generation. In one study [17], ChatGPT-5 was prompted to generate realistic portrayals of anorexia nervosa, bulimia nervosa, and binge-eating disorder. Initial outputs produced clichéd and highly stereotypical representations of these diagnoses: all images presented young, White women, often with narrow and appearance-focused visual cues that reinforced familiar assumptions about what people with eating disorders look like (e.g., emaciation for anorexia nervosa). However, a subtle change to the prompt substantially altered the outputs, such that when the model was asked to avoid clichéd stereotypes, the generated images became considerably more inclusive, showing greater variation in age, gender, race, and body shape across diagnostic categories [17]. These findings are noteworthy because they suggest that common LLMs may rely on stereotypical templates by default when visually representing complex psychiatric conditions, with diversity appearing only when explicitly requested. The impact of such outputs on population-level mental health literacy and awareness remains unknown, but default representations could reasonably be expected to perpetuate bias by narrowing public perceptions of how these illnesses present.

Overall, early evidence suggests that LLMs may support eating disorder education by providing accurate responses to some consumer-facing questions and generating more inclusive representations when prompted carefully. However, this evidence is scant and remains confined to a couple of small studies. Whether LLM-generated education improves understanding, reduces stigma, or supports timely help-seeking in real-world contexts remains unknown, but these are important questions for future research.

## Detection and Assessment

LLMs could also play a role in eating disorder detection and assessment, where their capacity to interpret language descriptions may help identify clinical symptoms and guide users to appropriate support. Such capabilities could have important implications across the healthcare pathway, allowing clinicians to use these tools as an additional source of decision support when considering differential diagnoses or referral options, or supporting patients to make sense of symptoms before entering treatment [18]. Demonstrations of these detection capabilities in the wider mental health field have mostly occurred in controlled settings, where researchers use structured clinical case vignettes to test whether LLMs can infer probable diagnoses or recommend appropriate management options [10]. These studies generally show that newer language models outperform earlier iterations in generating correct diagnoses and recommending appropriate treatments [19], with some structured evaluations reporting performance that exceeds human clinicians [20]. Beyond clinical vignettes, LLMs have also been trialled for assessment development, with one study showing that ChatGPT-4 could generate a new depression and anxiety screening questionnaire that demonstrated acceptable reliability and convergence with established PHQ-9 and GAD-7 measures [21].

In eating disorders, initial evidence points to the potential of LLMs to aid illness detection by identifying likely diagnoses from clinical case vignettes. Linardon and Messer [22] evaluated ChatGPT-5.4 using two structured vignettes: one depicting a restrictive eating disorder across low-, average-, and higher-weight presentations, manipulated to examine potential weight-related biases in detection, and another depicting binge-eating disorder. For the restrictive eating disorder vignette, ChatGPT correctly identified an eating disorder in 90%-100% of responses and always recommended specialist treatment, compared with 16%-47% and 17%-35% of clinician responses, respectively. For the binge-eating disorder vignette, ChatGPT correctly identified the diagnosis in all prompt repetitions, compared with 59% of community participants, and more consistently endorsed evidence-based treatment (100% vs. 50%). These patterns were replicated in sensitivity analyses using Claude-4.5 and Gemini-3 models [22]. In another study [23], ChatGPT-5.2 and Gemini 2.5 were tested using a case description indicative of anorexia nervosa and were asked to generate a diagnostic interpretation and therapeutic approach. Both models correctly identified anorexia nervosa as the probable diagnosis, distinguished it from other eating disorder presentations, and produced structured formulations linking weight loss, restrictive eating, excessive exercise, and cognitive distortions to appropriate psychological and nutritional treatment targets [23]. While informative, this study should be interpreted cautiously because it relied on qualitative assessment of single-session outputs rather than formal scoring or repeated testing.

Beyond individual-level diagnostic identification, LLMs could also support scalable assessment of eating disorder risk signals in online environments, where the volume and complexity of user-generated text exceed the capacity for manual review. For example, Chu et al. [24] collected 2.6 million tweets related to eating disorders, dieting, weight loss, and body image, identified distinct online communities based on patterns of retweet interaction, and fine-tuned Llama-3 models to represent each community’s language patterns. The models were then administered items from a validated eating disorder screening scale, with the model aligned to the pro-eating disorder community scoring considerably higher on eating disorder risk indicators than the other community-aligned models [24]. These findings suggest that LLMs could support large-scale eating disorder surveillance by translating unstructured online discourse into clinically relevant markers of risk that are not readily observable through conventional assessment methods. In practice, this could help identify harmful digital environments earlier and enable more targeted prevention efforts.

This research overall suggests that LLMs may have useful capabilities for eating disorder detection and assessment, from identifying likely diagnoses in structured case descriptions to assessing risk signals in online environments. However, because these findings are based either on hypothetical vignettes, single-session prompts, or community-level simulations, the effectiveness of LLMs in real clinical encounters with complex clinical presentations remains unknown. These limitations are compounded by evidence that consumer-facing models may produce biased eating disorder assessments, for example, assuming sex differences in perceived impairment despite otherwise comparable case descriptions [25]. This highlights the need for more rigorous research to better define the capabilities, limits, and safeguards required for LLM-supported eating disorder detection and assessment before any clinical deployment.

## Intervention

The ability to deliver mental health interventions at scale is perhaps the most ambitious clinical application of LLMs. The ease with which these tools can be accessed at low cost has the potential to reach many more people who might not otherwise seek help through traditional channels. Yet unlike earlier digital mental health tools that rely on static psychoeducational content or predetermined modules, LLMs can instantly adapt their responses to a user’s immediate concerns and simulate a more personalized therapeutic exchange [26]. For individuals showing early risk markers, LLMs could potentially intervene before symptoms become entrenched by delivering tailored preventative strategies in response to emerging concerns and directing users toward additional support when self-management of risk is no longer sufficient. These unique capabilities have led to a growing body of research evaluating LLMs across numerous intervention-relevant tasks in mental health, including the ability to respond empathically to distress [27], recommend therapeutic strategies [28], simulate counselling dialogue [29], and guide users through self-help exercises [30].

There is evidence to suggest that general-purpose and domain-specific LLMs can act as supportive agents, capable of responding to eating disorder symptom concerns with empathy, practical guidance, and clinically appropriate content. For example, one study prompted ChatGPT-4o with 32 bulimia nervosa-related symptom concerns and evaluated its capacity to provide emotional, informational, appraisal, and instrumental support [31]. Emotional support reflected empathy and compassion; informational support involved advice, behavioural strategies, and suggested actions; appraisal support provided feedback or affirmation to aid self-evaluation; and instrumental support referred to tangible assistance or access to services. Responses were rated by mental health professionals using a five-point scale (1 = poor, 5 = excellent), with ChatGPT performing strongest for informational support (M = 5.0, SD = 0) and also highly for emotional support (M = 4.3, SD = 0.7) and appraisal support (M = 4.3, SD = 0.6), but less well for instrumental support (M = 1.9, SD = 0.3). The authors suggest that general-purpose LLMs may provide complementary social support for people with bulimia nervosa, especially through psychoeducation, validation, and reflective guidance [31]. More specialized support may be possible when LLMs are embedded within domain-specific systems that are trained to adhere to evidence-based treatments. Hanzawa et al. evaluated a domain-specific AI agent designed to provide family-based therapy (FBT)-concordant guidance to caregivers of young people with anorexia nervosa. Across 477 hypothetical caregiver queries reflecting common FBT challenges, 92% of responses were rated clinically appropriate, including 92% for FBT-related questions, 88% for general anorexia nervosa knowledge, and 94% for parental distress [31]. These findings suggest that domain-specific systems may be able to extend evidence-based support beyond scheduled treatment sessions by helping families navigate the challenges of implementing FBT in daily life.

However, findings like these need to be interpreted alongside an emerging body of evidence showing that LLM outputs can introduce clinically relevant risks in eating disorders [32]. One study analysed responses generated by four general-purpose LLMs to 3,634 Reddit eating disorder community posts, identifying several potential harms from model outputs, including reinforcement of eating disorder behaviours, implicit weight-related bias, insensitivity to emotional cues, and overgeneralised health advice [33]. Further investigations suggest that risks like these may arise from models’ tendency to generate “food noise,” defined here as ostensibly supportive content that reinforces preoccupation with calories, portion regulation, and rigid healthy-eating rules [34]. Indeed, LLM outputs rated as definitionally consistent with “food noise” reached as high as 60% in one study [34], with these rates being most pronounced in high-risk scenarios involving fictitious users interacting with the model in ways consistent with a clinical eating disorder. Harms like these may also extend to real users, with pilot evaluations of LLM-based eating disorder chatbots showing that participants reported receiving context-insensitive validation, avoidance-based coping strategies, and unsupported therapeutic advice [35].

The findings reviewed above clearly highlight the need for more stringent evaluation of LLM-based eating disorder interventions in real users, ideally progressing from small-scale proof-of-concept designs that collect rich user experience data to larger efficacy trials that quantify treatment effects. The pilot evaluation of an LLM-based *WellnessBot* in 26 adults with eating disorders provides a useful example of this early-stage approach, combining detailed user engagement data, quantitative evidence of within-group symptom reduction over time, and qualitative feedback on experiences of interacting with the chatbot [35]. Larger randomized trials, ideally with active comparator conditions, should be the objective if claims are to be made about the clinical benefit of LLM-based interventions. The landmark trial evaluating *Therabot* – a purpose-built generative AI chatbot for mental health intervention – provides important foundational evidence, showing 8-week reductions in weight concerns relative to a waitlist control in adults with major depression, generalized anxiety, or clinically high risk for feeding and eating disorders [36]. These findings are particularly relevant to prevention, as participants in the feeding and eating disorder subgroup were at clinically elevated risk but did not have an established eating disorder, suggesting that LLM-based support may help reduce risk before symptoms progress to a diagnosable condition. While encouraging, independent replication, evidence of sustained improvement, sampling clinically relevant populations, and trials using active comparison groups are needed to better understand the clinical potential of these tools in eating disorder treatment.

## Risks Associated with LLMs

The promise of LLMs in eating disorder care needs to be considered alongside the possibility that these systems may also introduce new forms of harm. Their responses can appear clinically credible even when they are incomplete or unsafe, and this is especially consequential in a population where subtle guidance can influence symptom trajectories. A central task for the field is therefore to define these risks more clearly and build safeguards that are proportionate to the contexts in which LLMs are used.

A key concern is that LLMs are capable of producing advice that sounds convincing even when it is wrong or clinically inappropriate. This is particularly problematic in eating disorders, where seemingly innocuous guidance about food or exercise can reinforce the psychopathology rather than support recovery. The withdrawal of the Tessa rule-based chatbot from the National Eating Disorder Association after reports that it provided harmful weight loss guidance illustrates how automated support can inadvertently conflict with recovery-oriented care [37]. For LLMs, systematic research should combine clear thresholds for acceptable responses with red-team testing designed to identify the conditions under which harmful outputs emerge and when the model should instead defer or escalate users to professional support.

Privacy and confidentiality are among the most prominent concerns raised by consumers and eating disorder clinicians and may ultimately constrain willingness to use LLMs in care [9]. These concerns are especially salient because users may disclose sensitive information without fully understanding how conversational data are stored or shared. The problem is compounded by the fact that many people may seek eating disorder support through general-purpose systems (e.g., ChatGPT) [9], which sit outside the clinical governance frameworks typically applied to dedicated mental health tools. This creates a regulatory blind spot in which systems can influence symptom interpretation and help-seeking without necessarily being held to equivalent standards for privacy or evidence [38]. Future research should examine how users understand and respond to LLM data practices, whether privacy concerns alter disclosure or uptake, and which technical or governance safeguards are sufficient to maintain trust in clinically sensitive interactions.

Over-reliance is a concern for both users and clinicians. Patients may come to treat an always-available LLM as a substitute for professional care, particularly when its conversational fluency creates an impression of expertise [18]. This could delay recognition of deterioration and timely access to appropriate clinical care. Clinicians face a parallel risk of automation bias, whereby LLM-generated assessments or treatment suggestions begin to exert undue influence on clinical judgement, even when they are incomplete or wrong [39]. This may not only lead to inappropriate treatment decisions but could erode the relational and empathic qualities central to effective therapy [8]. Understanding when LLM support augments rather than displaces human judgement, alongside developing training that equips clinicians to critically appraise, override, and communicate LLM-generated recommendations without weakening the therapeutic relationship, will be necessary.

These risks are likely to represent only part of a broader safety landscape that will continue to evolve as LLMs become more deeply embedded in mental healthcare. Other recognised concerns include model drift after software updates, inappropriate responses during crises, uncertainty around professional accountability, and the potential to widen existing disparities in access or care [18]. Across these risks, human clinical oversight should be viewed not as a fallback safeguard but as part of the safety architecture itself [8], especially in instances where LLM outputs influence how symptoms are interpreted or what treatment is sought. Defining where clinician involvement is essential, and where greater autonomy may be acceptable, will be important for responsible implementation.

## Future Directions and Concluding Remarks

The evidence base on LLMs in eating disorders remains small, but nascent research highlights their potential to support education, detection and assessment, and intervention. Table 1 summarizes a non-exhaustive list of ideas for future research that could accelerate our understanding of the capabilities, limits, and safety requirements of these models before they can be responsibly deployed in clinical eating disorder settings.

**Table 1:** Avenues for future research on large language models in eating disorders and methodological considerations

Domain	Future research idea	Methodological considerations
Education	◾Test accuracy, appropriateness, and safety across the full range of eating disorder presentations. ◾Evaluate whether LLM education improves literacy, symptom recognition, stigma, help-seeking, and knowledge retention. ◾Compare LLM-generated education against existing psychoeducational resources. ◾ Examine whether text and image outputs reinforce stereotypes. ◾ Evaluate whether LLMs can support clinician education and student training. ◾ Assess risks of over-reliance on LLMs for eating disorder education.	◾ Test prompts across diagnoses, symptom severity, demographic groups, and levels of ambiguity or emotional distress. ◾ Include user-level outcomes such as comprehension, recall, perceived credibility, trust, behavioural intentions, and subsequent help-seeking. ◾Benchmark LLM outputs against expert-developed materials and clinical guidelines. ◾ Use clinician, lived-experience, and consumer ratings to assess inclusiveness, safety, and actionability. ◾ Test performance in clinician-assisted, student-training, and non-specialist workforce contexts. ◾Examine the effects of prompt wording, guardrails, and clinician-assisted interpretation on output quality and user understanding.
Detection/Assessment	◾ Test whether LLMs can identify eating disorder symptoms, probable diagnoses, severity, and referral needs across diverse clinical presentations.	◾Use validated clinical vignettes, real-world patient narratives, and clinician-rated case materials that vary by diagnosis, severity, comorbidity, and risk.
	◾Determine whether promising vignette-based performance generalises to complex, ambiguous, and evolving real-world presentations.	◾ Move beyond single-prompt evaluations by testing repeated prompts, multi-turn interactions, and uncertainty in user-provided information.
	◾ Examine whether LLM assessment improves clinical decision-making, referral quality, or early detection compared with usual assessments. ◾ Investigate bias in LLM-generated assessments across weight status, gender, age, race, socioeconomic position, and diagnostic presentation. ◾ Clarify the appropriate clinical role of LLMs in assessment, including whether they should function as screening aids, decision-support tools, or referral-navigation supports rather than diagnostic authorities.	◾ Compare LLM-assisted assessment with clinician-only assessment, standard screening tools, and existing digital triage systems using clinically meaningful outcomes. ◾ Use manipulated case materials to test whether equivalent symptoms receive different diagnostic/severity ratings or referral recommendations across demographic groups. ◾ Define acceptable performance thresholds, escalation criteria, audit procedures, and human oversight requirements before any use in clinical or public-facing assessment settings.
Intervention	◾ Test whether LLM interventions can safely deliver eating disorder support across different diagnoses and symptom profiles. ◾Determine whether LLMs produce improvements in eating disorder symptoms, impairment, help-seeking, or treatment engagement. ◾Examine whether domain-specific systems produce safer and more clinically appropriate support than general-purpose LLMs. ◾ Evaluate whether LLMs can extend care between sessions by supporting self-monitoring, caregiver support, and treatment adherence. ◾ Clarify which users are most likely to benefit from LLM-based interventions and which users may require higher levels of clinical support.	◾ Begin with small-scale proof-of-concept studies that collect detailed engagement, usability, acceptability, safety, and user experience data. ◾ Progress to adequately powered randomized trials with active comparator conditions, longer follow-up. ◾ Compare general-purpose models with domain-specific systems that use curated knowledge bases, treatment-specific guardrails, escalation rules, and human oversight. ◾Test LLMs as adjuncts to established treatments, with outcomes capturing both symptom change and effects on clinician workload, therapeutic alliance, and treatment fidelity. ◾ Plan for testing theoretically relevant moderators of change in comparative efficacy trials.

Across the three domains, future research should ideally progress from controlled, proof-of-concept evaluations towards studies that test real-world utility. For education, near-term work should extend accuracy and reproducibility testing across a broader range of clinical presentations, before examining whether LLM-generated information improves user outcomes or supports trainee clinician education. For detection and assessment, near-term research should test whether promising vignette-based findings extend to real clinical presentations without introducing demographic or diagnostic bias, paving the way for comparative studies that evaluate whether LLM-assisted assessment improves decision-making relative to clinician-led assessment alone. For intervention, the field requires a staged evaluation pathway, beginning with feasibility, acceptability, usability, and safety studies before progressing to adequately powered comparative efficacy trials [7, 40].

Beyond patient-facing applications, an adjacent research agenda is needed for clinician-facing uses of LLMs. The key question is not simply whether these tools can generate clinical case notes, differential diagnoses, treatment plans, or training feedback, but whether they improve the efficiency and quality of clinical work once error correction and workflow disruption are considered [41]. Studies should evaluate both proximal outcomes (e.g., documentation time, decision accuracy, treatment fidelity, clinician cognitive load, etc.) and downstream consequences for patient-facing care, including whether time saved is genuinely redirected toward therapeutic contact and whether LLM-assisted decisions improve care quality. This will require prospective evaluations embedded within routine services, with careful attention to when clinician oversight adds value, when automation introduces new risks, and which tasks are appropriate to delegate at all.

Given their accessibility and rapidly expanding capabilities, LLMs are likely to transform how eating disorders are managed. The field is now at a critical inflection point, where the evidence, safeguards, and evaluation standards established today will influence how these tools are used in practice tomorrow. Early adoption among eating disorder clinicians and community members highlights the need for coordinated research that addresses the most pressing questions about LLMs’ clinical capabilities. This paper provides a starting point by outlining early signals of promise and risks, and offering avenues for future research that will help ensure that these technologies deliver genuine clinical benefit for people with eating disorders.

## Key References

- Linardon J, Liu C, Messer M, McClure Z, Anderson C, Jarman HK. Current Practices and Perspectives of Artificial Intelligence in the Clinical Management of Eating Disorders: Insights From Clinicians and Community Participants. International Journal of Eating Disorders: 2025;58:724–34.
  - ○ Foundational evidence showing that eating disorder clinicians are using LLMs for administrative support, while community members are using them for education and self-help purposes.
- Yeşilkaya C, Keleş HK, Taşpolat ER, Mutlu C, Turan S. A Comparative Evaluation of Three Large Language Models for Parent‐Centered Questions About Anorexia Nervosa. International Journal of Eating Disorders: 2026.
  - ○ This study provides one of the first eating disorder-specific evaluations of general-purpose LLMs for consumer education, showing that three LLMs could provide generally accurate and reproducible responses to parent-centred questions about adolescent anorexia nervosa.
- Linardon J, Messer M. Benchmarking Generative Artificial Intelligence Against Human Judgment in Eating Disorder Case Recognition and Treatment Recommendations. International Journal of Eating Disorders: 2026.
  - ○ This study directly benchmarks LLM performance against human judgment, showing that ChatGPT-5 may identify eating disorder presentations and recommend evidence-based treatment more consistently than clinicians or community participants in structured case vignettes.
- Schnepper R, Roemmel N, Schaefert R, Lambrecht-Walzinger L, Meinlschmidt G. Exploring biases of large language models in the field of mental health: comparative questionnaire study of the effect of gender and sexual orientation in anorexia nervosa and bulimia nervosa case vignettes. JMIR Mental Health: 2025;12(1):e57986.
  - ○ This study shows that LLM-generated eating disorder assessments can demonstrate possible gender biases, with inferences to functional impairment varying by gender despite otherwise comparable case vignettes.
- Winecoff A, Klyman K, editors. From Symptoms to Systems: An Expert-Guided Approach to Understanding Risks of Generative AI for Eating Disorders. Proceedings of the 2026 CHI Conference on Human Factors in Computing Systems; 2026.
  - ○ This study develops an expert-informed, layered taxonomy of generative AI risks for eating disorders, showing how harms may arise not only from individual model outputs but also from broader system design, user interaction, and sociotechnical contexts.
- Choi R, Kim T, Park S, Kim JG, Lee S-J, editors. Private yet social: How LLM chatbots support and challenge eating disorder recovery. Proceedings of the 2025 CHI Conference on Human Factors in Computing Systems; 2025.
  - ○ This pilot study provides real-user evidence on how LLM chatbots may support eating disorder recovery through private and accessible interactions, while also showing that users may place trust in responses that contain clinically problematic guidance.

## Data Availability

No datasets were generated or analysed during the current study.
